# Erratum to “Flammability testing of 22 conventional European pediculicides”

**DOI:** 10.1007/s00436-017-5637-1

**Published:** 2017-10-02

**Authors:** Frank Eertmans, Bart Rossel

**Affiliations:** Oystershell Laboratories, Drongen, Belgium

Dear Editor,

We have read with great interest the publication of Dr. Dörge in *Parasitology Research* (2017; 116(4): 1189–1196) (10.1007/s00436-017-5396-z) regarding the flammability of 22 conventional European pediculicides. Our attention is drawn to some important issues in the article that require rectification and dissemination.

1. The opposite outcomes of the tests with Prioderm Shampoo Plus (rapid ignition, declining burn-off) and Elimax shampoo (heavily delayed ignition) are problematic since both products have the same composition, sold under two different brand names. This puts the ignition methods used by the authors in question. Perhaps the method is too variable to distinguish degrees of ignition (due to different hair strands, amount of product applied…)?

2) In Table 1, it is noted that there is no indication of flammability on the packaging of Elimax shampoo. However, the original instructions for use (present in the packaging, tested by the authors) include the following warning: “Keep treated hair away from naked flames.” Additionally, since 2017, emphasized warnings and symbols are on the pack or leaflet: “Keep treated hair away from fire, flames or hot objects (candles, fireplace, hair dryer, stove….” and “Never smoke during use or treatment.” This warning is common to the four products, developed by Oystershell and included in the publication (Elimax shampoo, Mosquito Läuse Shampoo 10, Mosquito Läuse Haarfluid, Prioderm Shampoo Plus).

3) Meda Pharma (Prioderm Shampoo Plus) and WEPA Apothekenbedarf (Mosquito products) are distributors but not the legal manufacturer, as mentioned in Table 1.

We would appreciate it if you would publish this rectification in *Parasitology Research*.

Best regards

Frank Eertmans

